# Differentiation between Primary Cerebral Lymphoma and Glioblastoma Using the Apparent Diffusion Coefficient: Comparison of Three Different ROI Methods

**DOI:** 10.1371/journal.pone.0112948

**Published:** 2014-11-13

**Authors:** Sung Jun Ahn, Hyun Joo Shin, Jong-Hee Chang, Seung-Koo Lee

**Affiliations:** 1 From the Department of Radiology, Severance Hospital, Yonsei University College of medicine, Seoul 120-752, Korea; 2 From the Department of Neurosurgery, Yonsei University College of medicine, Seoul 120-752, Korea; NIH, United States of America

## Abstract

**Objective:**

Apparent diffusion coefficients (ADC) can help differentiate between central nervous system (CNS) lymphoma and Glioblastoma (GBM). However, overlap between ADCs for GBM and lymphoma have been reported because of various region of interest (ROI) methods. Our aim is to explore ROI method to provide the most reproducible results for differentiation.

**Materials and Methods:**

We studied 25 CNS lymphomas and 62 GBMs with three ROI methods: (1) ROI_1_, whole tumor volume; (2) ROI_2_, multiple ROIs; and (3) ROI_3_, a single ROI. Interobserver variability of two readers for each method was analyzed by intraclass correlation(ICC). ADCs were compared between GBM and lymphoma, using two-sample *t*-test. The discriminative ability was determined by ROC analysis.

**Results:**

ADCs from ROI_1_ showed most reproducible results (ICC >0.9). For ROI_1_, ADC_mean_ for lymphoma showed significantly lower values than GBM (p = 0.03). The optimal cut-off value was 0.98×10^−3^ mm^2^/s with 85% sensitivity and 90% specificity. For ROI_2_, ADC_min_ for lymphoma was significantly lower than GBM (p = 0.02). The cut-off value was 0.69×10^−3^ mm^2^/s with 87% sensitivity and 88% specificity.

**Conclusion:**

ADC values were significantly dependent on ROI method. ADCs from the whole tumor volume had the most reproducible results. ADC_mean_ from the whole tumor volume may aid in differentiating between lymphoma and GBM. However, multi-modal imaging approaches are recommended than ADC alone for differentiation.

## Introduction

Glioblastoma (GBM) is the most common malignant brain tumor in adults. GBM is marked by rapid growth [1]. Primary central nervous system(CNS) lymphoma is less common than GBM but its incidence is increasing [2]. For GBM, surgical resection is the primary treatment [3], while chemotherapy or radiation therapy is the treatment of choice for CNS lymphoma [4]. Therefore, an exact differential diagnosis is essential for making therapeutic decisions about GBM and CNS lymphoma. On conventional imaging, primary CNS lymphomas usually show homogenous and intense contrast enhancement. And primary CNS lymphomas are often hypointense to gray matter without large necrosis on T2-weighted image(T2WI) [5]. However, differentiation is often difficult because some of GBMs have considerable overlap in conventional magnetic resonance(MR) imaging findings [6].

Several studies have shown that apparent diffusion coefficient (ADC) values from diffusion-weighted imaging(DWI) can help differentiate between CNS lymphoma and GBM [7]–[9]. However, other studies have reported that ADC might not be helpful because of substantial overlap between values for CNS lymphoma and GBM [10], [11].

These contradictory results are partly because ADC can be measured by a variety of methods to determine placement of the region of interest (ROI). Toh et al [9] drew the ROI in the center of the solid enhancing region Yamashita et al [12] and Doskaliyev et al [13] drew several small ROIs within the tumor. This might contribute to the wide variety in reported ADC results. Thus, it is necessary to evaluate the reliability of commonly used ROI methods in DWI. The purpose of this study was to compare whole tumor volume ROI, multiple ROIs and single ROI for ADC measurement for differentiating between primary CNS lymphoma and GBM.

## Materials and Methods

### Patients

Approval by Severance hospital institutional review board was obtained and informed consent was waived for this retrospective study. Patients' records and information were anonymized and de-identified prior to analysis. MR imaging of consecutive patients from Oct 2012 through Nov 2013 were retrospectively analyzed. We identified 30 immunocompetent patients with biopsy-proven primary CNS lymphoma. We excluded the 5 patients with primary CNS lymphoma because they received the steroid therapy before they performed MR imaging. Finally, 25 patients with primary CNS lymphoma (15 women, 10 men; mean age, 60 years; age range, 44–77 years) were included. We identified 62 patients (28 women and 34 men;mean age, 56.72 years; age range, 32–73 years) with histologically-confirmed, World Health Organization grade IV GBM in our medical record.

### MR imaging

All images were obtained using a 3.0T MRI scanner (Achieva, Philips Medical system, Best, Netherlands) with a 16-channel sensitivity encoding (SENSE) head coil. Diffusion weighted image(DWI) was performed using a single-shot spin-echo (SE) echo planar sequence with following parameters: Echo time(TR)/Repetition time(TE) = 8413/77 ms, 90° flip angle, 70 transverse sections, SENSE factor = 2, slice thickness = 2 mm, 112×112 matrix, field of view(FOV) = 220 mm. Diffusion-sensitizing gradients were applied sequentially in the x, y and z directions with b factors of 0 and 1000 s/mm^2^. ADCs were automatically calculated by the operating console of the MR scanner and displayed as corresponding ADC maps.

Postcontrast T1-weighted 3D-gradient echo sequence(GRE) imaging was obtained with following parameters: TR/TE = 9.86/4.59 ms, flip angle, 8°, 224×224 matrix with 224 phase-encoding steps; 1-mm section thickness; and 220 mm FOV. A standard dose (0.1 mmol/kg body weight) of gadoteric acid (Gd-DOTA, Dotarem; Laboratoire Guerbet, Aulnay-sous-Bois, France) was injected intravenously. Routine anatomic precontrast T1/T2 images were also obtained.

### Image analysis

**Figure 1:**
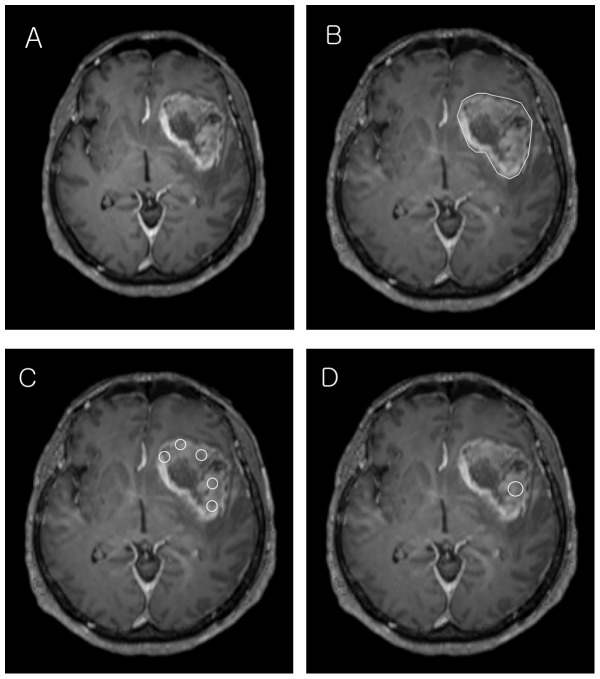
Representative case. A 43-year-old female with biopsy proven GBM. On Gd enhanced T1-weighed image (A), GBM shows heterogeneous enhancement in the left basal ganglia. ADC maps were coregistered to Gd enhanced T1-weighted image. Whole tumor volume ROI (ROI_1_) was drawn along the tumor border for each consecutive slice of the coregistered image (B). Multiple circular ROIs (ROI_2_) were drawn on coregistered image (area = 10 mm^2^) (C). A single circular ROI (ROI_3_) was drawn on any solid area, avoiding necrosis (area = 20 mm^2^) in coregistered image (D).

The size and location of tumor was recorded by the study coordinator. If there were multiple lesions, the largest one was measured. Three different ADC measurements for one lesion were obtained from the ADC map according to three distinct ROI protocols: (1) whole tumor volume; (2) multiple ROIs and (3) single ROI. For whole tumor volume, using the coregistration module integrated in the commercial software nordicICE (Nordic Imaging Lab, Bergen, Norway), ADC maps were coregistered to postcontrast T1-weighted 3D GRE image by the study coordinator. Two readers (a neuroradiologist with 5 years of experience and a neuroradiologist with 14 years of experience) independently drew freehand ROIs along tumor borders on coregistered images to cover tumors completely with consecutive slices. Minimum, maximum and mean value (min, max and mean) were calculated from ADC values from the whole tumor volume. For multiple ROIs, two readers independently drew circular 5 ROIs (area = 10 mm^2^) on enhancing lesions in coregistered ADC map. For single ROI method, the readers reviewed the coregistered ADC maps and drew a single circular ROI (area = 20 mm^2^) on any enhancing portion. Hemorrhage, cyst and necrosis were avoided when drawing all three ROI methods (Fig. 1). Min, max and mean were calculated as above.

### Statistical analysis

Statistical analyses were performed using SPSS version 16.0 (SPSS Inc., Chicago, IL, USA). Interobserver variability of the readers for different ROI methods was calculated as intraclass correlation(ICC) coefficient (0.00–0.20 poor, 0.21–0.40 fair, 0.41–0.60 moderate, 0.61–0.80 good and 0.81–1.00 excellent correlation). ADCs were averaged between the two observers for further analysis. ADC_min_, ADC_max_ and ADC_mean_ were compared between GBM and lymphoma using a two-sample *t*-test for each individual ROI method. Sensitivity, specificity and accuracy for the discriminating between GBM and lymphoma were calculated for each parameter using an optimal cut-off value determined by receiver operating characteristic (ROC) analysis. Area-under-the-ROC curve (AUC) values for discrimination were calculated for the four parameters. P-values<0.05 were considered statistically significant.

## Results

The most frequent location of primary CNS lymphoma was the cerebral hemisphere (13 out of 25, 52%), followed by the corpus callous (7 out of 25, 26%), deep nuclei (4 out of 25, 18%) and deep white matter (1 out of 25, 4%). The mean size of primary CNS lymphoma was 26.4 mm (range, 16∼54 mm). The most frequent location of GBM was the cerebral hemisphere (34 out of 62, 55%), followed by the deep nuclei (12 out of 62, 20%), corpus callosum (9 out of 62, 14%) and deep white matter (7 out of 62, 11%). The mean size of GBM was 30.3 mm (range, 9∼50 mm).

### Interobserver variability

Intraclass correlation coefficients between two readers for three ROI methods are in Table 1. ADC_min_, ADC_max_, ADC_mean_ from whole tumor volumes showed excellent interobserver reproducibility (ICC = 0.94, 0.92, 0.96 respectively). ADC_min_, ADC_max_, ADC_mean_ obtained from multiple ROIs showed good to excellent interobserver reproducibility (ICC = 0.86, 0.81, 0.78 respectively). ADC_min_, ADC_max_, ADC_mean_ from ROI_3_ showed good interobserver reproducibility (ICC = 0.69, 0.74, 0.72 respectively).

**Table 1:** Interobserver variability measured as intraclass correlation coefficient for different ROI protocols.

	ROI protocols		
	ROI_1_	ROI_2_	ROI_3_
ADC_min_(10^−3^ mm^2^/s)	0.94	0.86	0.69
ADC_max_	0.92	0.81	0.74
ADC_mean_	0.96	0.78	0.72

ROI, region of interest; ADC, apparent diffusion coefficients;min, minimum; max, maximum.

Number presents intraclass correlation coefficients: 0.00–0.20, poor; 0.21–0.40, fair; 0.41–0.60, moderate; 0.61–0.80, good; 0.81–1.00, excellent correlation.

ROI_1_ indicates whole tumor volume; ROI_2_, multiple ROIs; ROI_3_, a single ROI method(any enhancing portion avoiding cyst).

### Comparison of GBM and lymphoma ADC variables

ADC measures for the three different ROI protocols are in Table 2. For ROI_1_, whole tumor volume, ADC_mean_ of lymphomas was significantly lower than ADC_mean_ for GBM ((0.87±0.18)×10^−3^ mm^2^/s vs. (1.28±0.24)×10^−3^ mm^2^/s, p = 0.03). However, differences in ADC_min_ and ADC_max_ were not significant between GBM and lymphoma (p>0.05). For ROI_2_, ADC_min_ was significantly lower for lymphoma than for GBM ((0.51±0.17)×10^−3^ mm^2^/s vs. (0.79±0.20)×10^−3^ mm^2^/s, p = 0.02). However, differences in ADC_max_ and ADC_mean_ were not significantly different between GBM and lymphoma (p>0.05). For ROI_3_, ADC variables were not significantly different between GBM and lymphoma (p>0.05).

**Table 2:** ADC variables for lymphoma and GBM using three different ROI methods.

Variable	Lymphoma	GBM	*p*
ROI_1_			
Min(10^−3^ mm^2^/s)	0.41±0.18	0.48±0.15	0.37
Max	2.16±0.53	2.45±0.64	0.24
Mean	0.87±0.18	1.28±0.24	0.03*
ROI_2_			
Min	0.51±0.17	0.79±0.20	0.02*
Max	1.02±0.24	1.04±0.28	0.34
Mean	0.73±0.20	0.85±0.17	0.25
ROI_3_			
Min	0.66±0.13	0.80±0.28	0.16
Max	0.91±0.20	0.98±0.25	0.47
Mean	0.79±0.15	0.89±0.25	0.25

ROI, region of interest; ADC, apparent diffusion coefficients;min, minimum; max, maximum; SD, standard deviation; GBM, glioblastoma.

ROI_1_ indicates whole tumor volume; ROI_2_, most enhancing portion; ROI_3_, conventional ROI method(any enhancing portion avoiding cyst).

*indicates statistical significance (*p*<0.05).

### ROC analysis

ADC variables from three different ROI methods were evaluated for discriminative ability using ROC analysis (Table 3). ADC _mean_ calculated from ROI_1_ was a significant predictor for differentiating lymphoma from GBM (p = 0.03). The optimal cut-off value was 0.98×10^−3^ mm^2^/s (sensitivity: 85%; specificity: 90%; AUC, 0.87). In ROI_2_, ADC_min_ was a significant predictor for differentiating lymphoma from GBM (p = 0.02). The optimal cutoff value was 0.72×10^−3^ mm^2^/s (sensitivity: 87%; specificity: 65%; accuracy: 0.84). Other variables from the three different ROI methods did not show significant discriminative ability (p>0.05).

**Table 3:** Sensitivity and specificity of ADC variables for differentiating lymphoma from GBM using ROC.

Variable	Cut off value	Sensitivity	Specificity	AUC	*p*
ROI_1_					
Min(10^−3^ mm^2^/s)	0.47	42	90	0.58	0.50
Max	2.52	41	92	0.63	0.28
Mean	0.98	85	90	0.87	0.01*
ROI_2_					
Min	0.69	87	88	0.84	0.02*
Max	1.04	85	45	0.64	0.21
Mean	0.83	50	90	0.70	0.06
ROI_3_					
Min	0.79	85	63	0.70	0.09
Max	1.07	85	54	0.59	0.44
Mean	0.90	85	54	0.64	0.24

ROI, region of interest; ADC, apparent diffusion coefficients; min, minimum; max, maximum; SD, standard deviation; GBM, glioblastoma; AUC, area-under-the-ROC curve.

ROI_1_ indicates whole tumor volume; ROI_2_, most enhancing portion; ROI_3_, conventional ROI method(any enhancing portion avoiding cyst).

*indicates statistical significance (*p*<0.05).

## Discussion

Previous studies have used various ROI methods to measure ADC values for differentiating between lymphoma and GBM [7]–[10]. Toh et al [9] drew a single ROI in the center of solid enhancing region and Yamashita et al [12] and Doskaliyev et al [13] drew several small ROIs. Kang et al [14] used the whole tumor volume ROI. These various ROI methods may account for previous inconsistent results. However, there has been no study comparing the reproducibility of various ROI selections. According to our results, interobserver reproducibility of ADC calculations was dependent on the selected ROI method. ADC measurements from the whole tumor volume (ROI_1_) were most reproducible followed by multiple ROIs, then by the single ROI method. Several studies reported that quantitative measurement from the whole tumor volume is the most reproducible, although their subjects was not the brain [15]–[17]. Our results suggested that the whole tumor volume ROI method is favored, and single ROI method should be avoided when measuring ADC values. A single ROI method can be subjective and prone to a sampling bias [18].

We found that the ADC_mean_ from the whole tumor volume was significantly lower for lymphoma than for GBM. Meanwhile, ADC_mean_ from multiple ROIs or a single ROI was not significantly different between lymphoma and GBM. It is well known that GBM may have heterogeneous histologic features. Although we draw ROIs avoiding large necrosis, GBM may have microscopic necrosis with surrounding clustered nuclei, so called “pseudopalisading” features, which may increase the overall ADC_mean_ [19], [20]. These features make it easier to differentiate between lymphoma and GBM. On the contrary, an ADC from multiple ROIs or a single ROI may not reflect heterogeneity of GBM [21].

Also of note was that ADC_min_ from the whole tumor volume was not a significant predictor but ADC_min_ from multiple ROIs was a significant predictor for differentiating between lymphoma and GBM. ADC_min_ has been suggested to reflect the highest tumor cell density or the most proliferative portion of a tumor within heterogeneous tumors. ADC_min_ from whole tumor volumes might be influenced by the susceptibility of MR to generate artifacts from blood products and might not represent true ADC_min_ of the tumor parenchyma.

**Figure 2:**
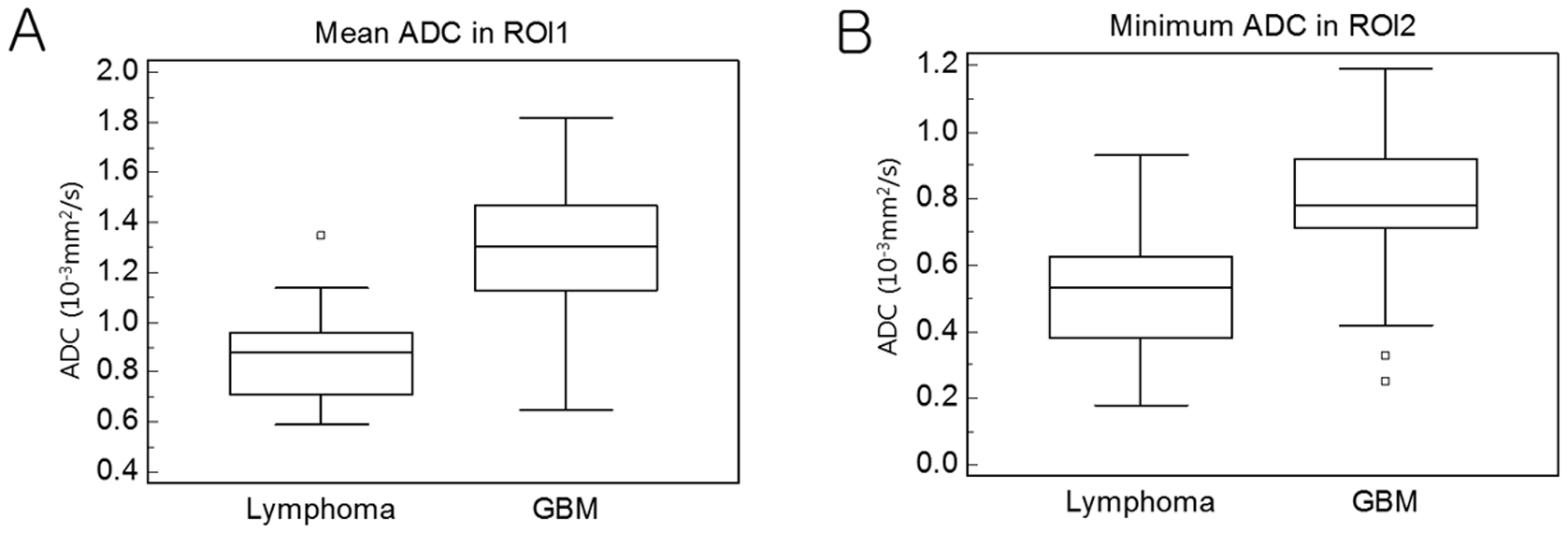
Box-and-whisker plots of representative ADC variables for lymphoma and GBM: mean ADC in ROI_1_ (A) and minimum ADC in ROI_2_ (B). The central box represents the value from the lower to upper quartile. The middle line represents the median. The horizontal line extends from the minimum to the maximum value. An outside value are plotted with s square marker.

However, our results should be carefully interpreted, because the ranges of ADCs between lymphoma and GBM still substantially overlapped (Fig. 2) and ADC alone might not be sufficient to differentiate lymphoma from GBM. Other advanced imaging techniques such as dynamic contrast-enhanced MRI (DCE), dynamic susceptibility-weighted imaging (DSC), susceptibility-weighted imaging (SWI) and FDG-PET have been reported to improve differential diagnosis of lymphoma and GBM [22]–[25]. Kickingereder et al [26] reported multimodal imaging integrating these advanced sequences allowed reliable differentiation of lymphoma and GBM. Therefore, Multiple advanced imaging techniques in conjunction with ADC should be preferred than ADC alone when differentiating lymphoma from GBM.

Our study has limitations. First, selection bias was ineluctable in this study because only the patients who had pathologically proven lymphoma and GBM were enrolled. Second, it was difficult to spatially co-localize pathology with MR images. Therefore, interpreting the pathological meaning of ADC from each ROI was difficult. Third, the number of cases was not enough to draw a solid conclusion. Fourth, we did not perform ADC histogram analysis and the distribution of ADCs were not assessed.

In conclusion, ADC values were significantly dependent on ROI method. ADCs from the whole tumor volume had the most reproducible results. ADC_mean_ from the whole tumor volume may aid in differentiating between lymphoma and GBM. However, multi-modal imaging approaches are recommended than ADC alone for the differentiation.

## References

[1] Stupp R, Mason WP, van den Bent MJ, Weller M, Fisher B, et al (2005) Radiotherapy plus concomitant and adjuvant temozolomide for glioblastoma. N Engl J Med 352: 987–996. 15758009 10.1056/NEJMoa043330

[2] Surawicz TS, McCarthy BJ, Kupelian V, Jukich PJ, Bruner JM, et al (1999) Descriptive epidemiology of primary brain and CNS tumors: results from the Central Brain Tumor Registry of the United States, 1990–1994. Neuro Oncol 1: 14–25. 11554386 10.1093/neuonc/1.1.14 PMC1919458

[3] Giese A, Westphal M (2001) Treatment of malignant glioma: a problem beyond the margins of resection. J Cancer Res Clin Oncol 127: 217–225. 11315255 10.1007/s004320000188 PMC12164938

[4] Batchelor T, Loeffler JS (2006) Primary CNS lymphoma. J Clin Oncol 24: 1281–1288. 16525183 10.1200/JCO.2005.04.8819

[5] Haldorsen IS, Espeland A, Larsson EM (2011) Central nervous system lymphoma: characteristic findings on traditional and advanced imaging. AJNR Am J Neuroradiol 32: 984–992. 20616176 10.3174/ajnr.A2171 PMC8013157

[6] Al-Okaili RN, Krejza J, Woo JH, Wolf RL, O'Rourke DM, et al (2007) Intraaxial brain masses: MR imaging-based diagnostic strategy–initial experience. Radiology 243: 539–550. 17456876 10.1148/radiol.2432060493

[7] Calli C, Kitis O, Yunten N, Yurtseven T, Islekel S, et al (2006) Perfusion and diffusion MR imaging in enhancing malignant cerebral tumors. Eur J Radiol 58: 394–403. 16527438 10.1016/j.ejrad.2005.12.032

[8] Yamasaki F, Kurisu K, Satoh K, Arita K, Sugiyama K, et al (2005) Apparent diffusion coefficient of human brain tumors at MR imaging. Radiology 235: 985–991. 15833979 10.1148/radiol.2353031338

[9] Toh CH, Castillo M, Wong AM, Wei KC, Wong HF, et al (2008) Primary cerebral lymphoma and glioblastoma multiforme: differences in diffusion characteristics evaluated with diffusion tensor imaging. AJNR Am J Neuroradiol 29: 471–475. 18065516 10.3174/ajnr.A0872 PMC8118870

[10] Batra A, Tripathi RP (2004) Atypical diffusion-weighted magnetic resonance findings in glioblastoma multiforme. Australas Radiol 48: 388–391. 15344992 10.1111/j.0004-8461.2004.01324.x

[11] Toh CH, Chen YL, Hsieh TC, Jung SM, Wong HF, et al (2006) Glioblastoma multiforme with diffusion-weighted magnetic resonance imaging characteristics mimicking primary brain lymphoma. Case report. J Neurosurg 105: 132–135. 16871888 10.3171/jns.2006.105.1.132

[12] Yamashita K, Yoshiura T, Hiwatashi A, Togao O, Yoshimoto K, et al (2013) Differentiating primary CNS lymphoma from glioblastoma multiforme: assessment using arterial spin labeling, diffusion-weighted imaging, and (1)(8)F-fluorodeoxyglucose positron emission tomography. Neuroradiology 55: 135–143. 22961074 10.1007/s00234-012-1089-6

[13] Doskaliyev A, Yamasaki F, Ohtaki M, Kajiwara Y, Takeshima Y, et al (2012) Lymphomas and glioblastomas: differences in the apparent diffusion coefficient evaluated with high b-value diffusion-weighted magnetic resonance imaging at 3T. Eur J Radiol 81: 339–344. 21129872 10.1016/j.ejrad.2010.11.005

[14] Kang Y, Choi SH, Kim YJ, Kim KG, Sohn CH, et al (2011) Gliomas: Histogram analysis of apparent diffusion coefficient maps with standard- or high-b-value diffusion-weighted MR imaging–correlation with tumor grade. Radiology 261: 882–890. 21969667 10.1148/radiol.11110686

[15] Lambregts DM, Beets GL, Maas M, Curvo-Semedo L, Kessels AG, et al (2011) Tumour ADC measurements in rectal cancer: effect of ROI methods on ADC values and interobserver variability. Eur Radiol 21: 2567–2574. 21822946 10.1007/s00330-011-2220-5 PMC3217149

[16] Goh V, Halligan S, Gharpuray A, Wellsted D, Sundin J, et al (2008) Quantitative assessment of colorectal cancer tumor vascular parameters by using perfusion CT: influence of tumor region of interest. Radiology 247: 726–732. 18403621 10.1148/radiol.2473070414

[17] Chalian H, Tochetto SM, Tore HG, Rezai P, Yaghmai V (2012) Hepatic tumors: region-of-interest versus volumetric analysis for quantification of attenuation at CT. Radiology 262: 853–861. 22357887 10.1148/radiol.11110106

[18] Tozer DJ, Jager HR, Danchaivijitr N, Benton CE, Tofts PS, et al (2007) Apparent diffusion coefficient histograms may predict low-grade glioma subtype. NMR Biomed 20: 49–57. 16986106 10.1002/nbm.1091

[19] Huang BC, Geng DY, Zee CS, Ji YM, Cheng HX, et al (2010) A unique magnetic resonance imaging feature of glioblastoma multiforme: the ‘pseudopalisade’ sign. J Int Med Res 38: 686–693. 20515584 10.1177/147323001003800233

[20] Rees JH, Smirniotopoulos JG, Jones RV, Wong K (1996) Glioblastoma multiforme: radiologic-pathologic correlation. Radiographics 16: 1413–1438 quiz 1462-1413. 8946545 10.1148/radiographics.16.6.8946545

[21] Cha S (2006) Update on brain tumor imaging: from anatomy to physiology. AJNR Am J Neuroradiol 27: 475–487. 16551981 PMC7976984

[22] Kim HS, Jahng GH, Ryu CW, Kim SY (2009) Added value and diagnostic performance of intratumoral susceptibility signals in the differential diagnosis of solitary enhancing brain lesions: preliminary study. AJNR Am J Neuroradiol 30: 1574–1579. 19461062 10.3174/ajnr.A1635 PMC7051626

[23] Kickingereder P, Sahm F, Wiestler B, Roethke M, Heiland S, et al (2014) Evaluation of microvascular permeability with dynamic contrast-enhanced MRI for the differentiation of primary CNS lymphoma and glioblastoma: radiologic-pathologic correlation. AJNR Am J Neuroradiol 35: 1503–1508. 24722313 10.3174/ajnr.A3915 PMC7964431

[24] Makino K, Hirai T, Nakamura H, Murakami R, Kitajima M, et al (2011) Does adding FDG-PET to MRI improve the differentiation between primary cerebral lymphoma and glioblastoma? Observer performance study. Ann Nucl Med 25: 432–438. 21404136 10.1007/s12149-011-0483-1

[25] Liao W, Liu Y, Wang X, Jiang X, Tang B, et al (2009) Differentiation of primary central nervous system lymphoma and high-grade glioma with dynamic susceptibility contrast-enhanced perfusion magnetic resonance imaging. Acta Radiol 50: 217–225. 19096950 10.1080/02841850802616752

[26] Kickingereder P, Wiestler B, Sahm F, Heiland S, Roethke M, et al (2014) Primary Central Nervous System Lymphoma and Atypical Glioblastoma: Multiparametric Differentiation by Using Diffusion-, Perfusion-, and Susceptibility-weighted MR Imaging. Radiology 272: 843–850. 24814181 10.1148/radiol.14132740

